# Effect of PEI25k/DOTAP/cholesterol liposomal formulation on the immunogenicity of SARS-CoV-2 spike protein-encoding DNA vaccine

**DOI:** 10.5114/bta/216301

**Published:** 2026-03-06

**Authors:** Indira P. Negari, Azka N. Khoerunnisa, Tarwadi, Anissa N. Sari, Sabar Pambudi, Irvan Faizal, Cindy M. Septani

**Affiliations:** 1 Research Center for Vaccine and Drugs, National Research and Innovation Agency (BRIN), Bogor, West Java, Indonesia; 2 Faculty of Military Mathematics and Natural Sciences, Univeritas Pertahanan Indonesia, Bogor, West Java, Indonesia; 3 Department of Biotechnology, Universitas Esa Unggul, Jakarta, Indonesia; 4 School of Bioscience, Technology, and Innovation, Atma Jaya Catholic University of Indonesia, Tangerang, Banten, Indonesia; 5 Research Center of Polymer Technology, National Research and Innovation Agency (BRIN), South Tangerang, Banten, Indonesia

**Keywords:** IL-6, immunogenic, liposome, SARS-CoV-2, TNF-α

## Abstract

**Background:**

The Coronavirus Disease 2019 (COVID-19) pandemic has highlighted the need to upgrade vaccine strategies. DNA vaccines are advantageous because of their stability and ease of large-scale production; however, they often require adjuvants to boost immune response. Liposomal adjuvants have emerged as effective immune response enhancers, offering efficient delivery and compatibility with biological systems. This study evaluated the immune response induced by a Severe Acute Respiratory Syndrome Coronavirus 2 (SARS-CoV-2) spike protein-encoding DNA vaccine formulated with various liposome adjuvants in mice.

**Materials and methods:**

The interleukin (IL)-6 and tumor necrosis factor-alpha (TNF-α) mRNA expression levels were analyzed by reverse transcription-quantitative polymerase chain reaction. Flow cytometry was conducted to characterize leukocyte populations and assess immune activation status. Five target cell surface markers were analyzed in this study: CD4^+^ T, CD8^+^ T, CD11b^+^, CD14^+^, and CD45^+^ leukocytes, along with their corresponding detected cell types.

**Result:**

Among the tested formulations, the PEI25k : DOTAP : cholesterol (2 : 1 : 1) combination showed the most significant immunostimulatory effect. This formulation induced the lowest expression levels of IL-6 (0.17 ± 0.01) and TNF-α (0.25 ± 0.01) mRNAs in the treatment groups; furthermore, flow cytometry revealed robust activation of both adaptive and innate immune subsets, with an increase in CD4^+^ (44.00 ± 1.00), CD8^+^ (40.00 ± 1.00), CD11b^+^ (50.00 ± 2.00), CD14^+^ (42.00 ± 2.000), and CD45^+^ (29.00 ± 1.00) T cell populations. These results demonstrate an integrated immune response characterized by strong T cell activation, myeloid engagement, and recruitment of a broad leukocyte population, indicating balanced immunity with reduced inflammation risk.

**Conclusions:**

The PEI25k : DOTAP : cholesterol (2 : 1 : 1) formulation has high potential as a liposome-based adjuvant for enhancing the immunogenicity of DNA vaccines. The findings of this study support its suitability for further development as a potential adjuvant candidate, particularly for improving DNA vaccine delivery and immunogenicity against SARS-CoV-2.

## Introduction

According to the World Health Organization update in February 2025, Coronavirus Disease 2019 (COVID-19), caused by the Severe Acute Respiratory Syndrome Coronavirus 2 (SARS-CoV-2) virus, has resulted in more than 777 million reported cases and 7 million deaths globally, making it one of the most widespread and lethal infectious diseases in recent history (Lin et al. 2025). Human-to-human transmission of SARS-CoV-2 is primarily mediated by the virus’s spike (S) protein or envelope spike, which is responsible for invading the host cell (Parry et al. 2023). Following its entry, the virus replicates in the cell, which induces immune cells to secrete cytokines (Kang et al. 2020; Darif et al. 2021). Therefore, developing a reliable immunization strategy to protect against SARS-CoV-2 infection is a critical need. Interleukin-6 (IL-6) and tumor necrosis factor-alpha (TNF-α) are the major pro-inflammatory mediators involved in regulating the immune response to viral pathogens, including SARS-CoV-2 (Ye et al. 2020; McElvaney et al. 2020; Gorham et al. 2020; Dhall et al. 2021; Chen et al. 2022). According to previous reports, elevated IL-6 and TNF-α levels are closely associated with disease severity in COVID-19, contributing to systemic inflammation, cytokine storm, and multi-organ damage in patients with severe disease (Schultheiß et al. 2022). These cytokines are primarily produced by activated macrophages, monocytes, and T cells in response to viral recognition by innate immune sensors such as Toll-like receptors (TLRs) (Ciesielska et al. 2021; Smaoui and Yahyaoui 2021). For vaccination, regulated stimulation of IL-6 and TNF-α is crucial to trigger early defense mechanisms and facilitate antigen processing, T cell activation, and development of long-lasting adaptive immunity (Tanaka et al. 2014; Schoenmaker et al. 2021). However, excessive or prolonged cytokine expression may result in adverse inflammatory outcomes (Medzhitov 2008; Newton and Dixit 2012; Tanaka et al. 2014; Pinti et al. 2016). Therefore, it is critical to evaluate the expression levels of IL-6 and TNF-α to better understand the balance between vaccine-induced protection and potential inflammation.

According to a previous study, over 10 billion doses of various COVID-19 vaccine platforms, such as mRNA-based, viral vector-based, and DNA-based vaccines, have been administered worldwide (Viana et al. 2022). Several DNA vaccines have received regulatory approval in veterinary medicine, targeting diseases such as West Nile virus infection in horses (Redding and Weiner 2009; Aida et al. 2021). In recent years, numerous prophylactic DNA vaccine candidates have also advanced to human clinical trials, including those developed for Ebola virus, Chikungunya, HIV, and Zika virus (Muthumani et al. 2016; Gary and Weiner 2020; Ledesma-Feliciano et al. 2023). ZyCoV-D, a DNA vaccine against SARS-CoV-2, demonstrated 67% efficacy in clinical trials conducted in India during the dominance of the Delta variant (Mallapaty 2021). Beyond viral pathogens, ongoing preclinical and clinical investigations extend DNA vaccine applications to non-viral targets such as *Pseudomonas aeruginosa, Staphylococcus aureus*, tuberculosis, and cancer (Patel et al. 2017; Lee et al. 2018; Jahanafrooz et al. 2020; Sefidi-Heris et al. 2020), thereby highlighting the broad versatility of DNA vaccine platforms. This underscores the benefits of DNA vaccines, including flexibility in delivering multiple antigens, capacity to stimulate humoral and cellular immune responses, inherent stability, favorable safety profile, and suitability for mass manufacturing (Hayat Khan 2013; Smith et al. 2020; Narayanan et al. 2022; Ledesma-Feliciano et al. 2023). Adjuvants, defined as supplementary compounds added to vaccines to efficiently stimulate and enhance immune activity, have a critical role in boosting the immunogenicity of DNA-based vaccines (Pulendran et al. 2021; Facciolà et al. 2022; Narayanan et al. 2022). Delivery system adjuvants, such as liposomes, have been successfully utilized in mRNA-based COVID-19 vaccines, achieving up to 95% efficacy in the BNT162b2 vaccine series (Fan et al. 2022), thus demonstrating the potential of liposomes as safe and effective vaccine adjuvants. Therefore, polymer-based liposome formulations are required for preparing such adjuvants. Polyethylenimine (PEI), a cationic polymer widely used as a gene carrier, has an amino-rich structure that provides high positive charge density and has the potential to enhance liposome stability and strengthen structural integrity (Liang et al. 2019; Dai et al. 2020; Sriwidodo et al. 2022). Based on previous studies, high-molecular-weight PEI polymers, such as PEI25k, can improve the specificity of gene delivery and possess high transfection efficiency (Song et al. 2012; Lu et al. 2020). However, PEI25k is inherently toxic, which limits its application as an adjuvant (Lu et al. 2020). To address this issue, PEI/DNA polyplexes are commonly conjugated with cholesterol (Song et al. 2012). Cholesterol, as a neutral lipid, can eliminate excess free PEI25k chains and influence how the PEI25k/cholesterol complex associates with cellular membranes, thereby reducing membrane damage and suppressing PEI25k-associated cytotoxicity (Song et al. 2012). The use of cationic lipids such as 1,2-dioleoyl-3-trimethylammonium propane (DOTAP) can remarkably improve the effectiveness and structural stability of liposomes by enabling electrostatic interactions with DNA (Miatmoko et al. 2023).

Previous studies have shown that immunity generated after SARS-CoV-2 vaccination involves lymphocytes such as CD4^+^ T cells, which secrete cytokines that modulate the activation and specialization of additional immune components, and CD8^+^ T cells, which exhibit cytotoxic activity by producing perforin and granzyme to trigger cell death in virus-infected targets. Additionally, CD45, a leukocyte marker expressed on almost all hematopoietic cells, is vital for signaling through antigen receptors in lymphocytes, thereby playing a regulatory role in both early and acquired immune functions (Schmid et al. 2018; Courtney et al. 2019; Tiyo et al. 2021). This vaccine activates early immune cells such as macrophages, dendritic cells, neutrophils, and natural killer (NK) cells, which subsequently trigger adaptive immune responses; these cells carry markers such as CD11b and CD14, which serve as co-receptors for lipopolysaccharide (LPS) detection, thus playing a crucial role in initiating innate immune signaling and facilitating pathogen clearance (Wu et al. 2019; Ciesielska et al. 2021; Tiyo et al. 2021). These markers reflect the complex cellular interplay underlying vaccine-induced immunity. In the present study, we evaluated how different liposome-based adjuvant formulations – consisting of PEI25k, DOTAP, and cholesterol – influence immune modulation when combined with a plasmid DNA encoding the SARS-CoV-2 spike protein. These formulations were evaluated for their ability to induce immune responses, particularly through the expression of IL-6 and TNF-α mRNAs and the activation of leukocyte populations. The findings of this study are expected to support the development of a liposome-based adjuvant for improving DNA vaccine delivery and immunogenicity against SARS-CoV-2 that aligns with global health strategies.

## Materials and methods

### Ethical statement

The animal procedures in this study received ethical clearance from the Health Research Ethics Committee of Dr. Cipto Mangunkusumo National General Hospital, Faculty of Medicine, Universitas Indonesia, Depok, Indonesia (Approval Number: KET-111/UN.F1/ETIK/PPM.00.02/2024) under protocol number 23-12-2078, in accordance with ethical standards and scientific guidelines. Female BALB/c mice (8 weeks old) were sacrificed through neck dislocation (Lofgren et al. 2019).

### Spike (S) protein cloning, expression, and purification

A plasmid containing the SARS-CoV-2 spike (S) protein gene (9254 bp), obtained by double digesting the recombinant pcDNA3.1 full-length spike plasmid with restriction enzymes *Xho* I and *Xba* I (Figure S1), was introduced into *Escherichia coli* TOP10 cells. The transformed *E. coli* TOP10 cells were grown in 1 l Luria-Bertani (LB) broth containing 5 g yeast extract (Sigma-Aldrich Inc., Burlington, MA, USA), 10 g NaCl (Sigma-Aldrich Inc.), and 10 g tryptone (Oxoid Ltd., Basingstoke, Hampshire, UK). A starter culture of the recombinant *E. coli* was initially cultivated in 10 ml selective medium containing ampicillin (Sigma-Aldrich Inc.) for 2–3 h; the culture medium volume was then scaled up to 1 l under shaking condition at 200 rpm for 16 h at 37°C. S protein plasmids were extracted using the alkaline lysis method, purified with the DNA-midi™ GT Plasmid DNA Purification Kit (iNtRON, Seongnam-si, Gyeonggi-do, Republic of Korea), and analyzed by electrophoresis with 1% agarose gel prepared in 1× TAE buffer; the gel was mixed with 6× loading dye and stained with ethidium bromide for 20 min.

### Vaccination of mice

Plasmid DNA (50 µg) containing the sequence for the SARS-CoV-2 spike (S) protein was dissolved in 100 µl PBS and combined with an equal amount (100 µl) of one of the following liposomal formulations: PEI25k : cholesterol (1 : 1), PEI25k : DOTAP (1 : 1), PEI25k : DOTAP : cholesterol (1 : 1 : 1), or PEI25k : DOTAP : cholesterol (2 : 1 : 1). The DNA-liposome mixtures were prepared in microtubes by gentle pipetting to ensure homogeneous complex formation. Freund’s adjuvant was used as the reference control. BALB/c mice were intramuscularly immunized in the rectus femoris muscle. Four injections were administered per mouse at the intervals of 2 weeks. Two weeks after the final booster dose, mice were euthanized, and their spleen tissues and peritoneal exudate cells were isolated for RT-qPCR and flow cytometry analysis, respectively.

### RT-qPCR

Total RNA was isolated from spleen tissues of BALB/c mice by using a modified protocol in RNase-free conditions to preserve RNA quality, employing GENEzol reagent (Geneaid Biotech Ltd., Taiwan). Total RNA concentration and purity were estimated using a Nanodrop spectrophotometer (Thermo Fisher Scientific, Waltham, MA, USA). Primer sequences for the target genes were generated using the NCBI Primer-Blast tool (https://www.ncbi.nlm.nih.gov/tools/primerblast/). RT-qPCR was performed using the SensiFAST™ SYBR No-ROX One-Step Kit (Meridian Bioscience, Cincinnati, OH, USA) in accordance with the manufacturer’s instructions. Gene expression analysis was conducted using the QuantStudio 5 Real-Time PCR System (Thermo Fisher Scientific). Thermal cycling parameters were as follows: initial incubation at ≥ 37°C for 15 min; enzyme inactivation at 95°C for 10 min; and 40 amplification cycles comprising denaturation at 95°C for 10 s, annealing and fluorescence acquisition at 60°C for 30 s, and extension at 72°C for 30 s. GAPDH served as the internal control to normalize gene expression levels (Ghulam et al. 2022). Relative cytokine expression levels were determined using the comparative Ct method (ΔCt), and the 2^(–ΔΔCt)^ formula was used to determine fold changes in gene expression (Schmittgen and Livak 2008). The respective primers used for IL-6, TNF-α, and GAPDH were as follows: 5′-GAGTCACAGAAGGAGTGGCTAAG-3′ (forward), 5′-ACCACAGTGAGGAATGTCCAC-3′ (reverse); 5′-GCAACTGGCAGAAGAGGCACTC-3′ (forward), 5′-GCAGGAATGAGAAGAGGCTGAGAC-3′(reverse); and 5′-CGTCTTCACCACCATGGAGA-3′ (forward), 5′-CGGCCATCACGCCACAGTTT-3′ (reverse).

### Flow cytometry

The following five target cell surface markers were used: CD4 (APC-conjugated: eBioscience, Cat. No. 17-0112, 1 : 800), CD8 (PE-conjugated: eBioscience, Cat. No. 12-0081, 1 : 400), CD11b (FITC-conjugated: eBioscience, Cat. No. 17-0041, 1 : 400), CD14 (FITC-conjugated: eBioscience, Cat. No. 11-0141, 1 : 100), and CD45 (PE-conjugated: eBioscience, Cat. No. 12-0451, 1 : 800) (Thermo Fisher), along with their corresponding detected cell types (Delmonte and Fleisher 2019; Goodwin et al. 2020). Next, 55 µl peritoneal exudate was prepared, and an antibody mixture with flow cytometry staining buffer was added to each microtube containing the sample to make up the final volume to 110 µl. The samples were incubated for 30 min at 2–8°C in the dark. The cells were then washed with 200 µl flow cytometry staining buffer containing PBS and bovine serum albumin, followed by centrifugation at 400–600 × *g* for 5 min at ambient temperature; subsequently, the supernatant was carefully removed. The incubation and washing steps were repeated twice before staining the cells with 7-AAD viability staining solution (Thermo Fisher Scientific). Samples were incubated for 5–15 min at 2–8°C and then measured by Attune NxT flow cytometer (Thermo Fisher Scientific) (Lin et al. 2025).

### Statistical analysis

The Shapiro-Wilk test was conducted to assess data normality. Data with normal distribution were analyzed with one-way ANOVA followed by Tukey’s post-hoc analysis; the results were visualized with GraphPad Prism software (San Diego, CA, USA). Different letters indicate significant differences between the groups (*p* < 0.05; *n* = 3). The average values and standard deviations were determined from a minimum of three independent experiments (Negari et al. 2021).

## Results

### IL-6 and TNF-α mRNA expression levels in mice spleen

**Figure 1:**
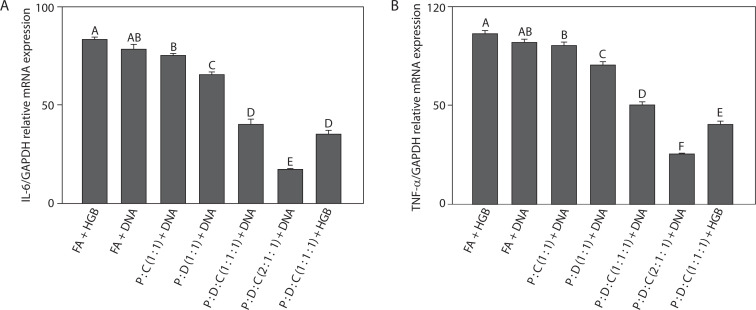
Reduction in the mRNA level expression. SARS-CoV-2 spike protein-encoding DNA vaccines formulated with the following liposomal formulations were administered to mice: PEI25k : cholesterol (1 : 1), PEI25k : DOTAP (1 : 1), PEI25k : DOTAP : cholesterol (1 : 1 : 1), and PEI25k : DOTAP : cholesterol (2 : 1 : 1). Post-vaccination, spleen tissues of mice from all the above mentioned groups were analyzed by RT-qPCR for the expression levels of (**A**) IL-6 and (**B**) TNF-α gene relative to the GAPDH gene. Different letters indicate significant differences between the groups (p < 0.05; n = 3)

To evaluate the inflammatory response induced by liposome-based adjuvant on SARS-CoV-2 spike protein-encoding DNA, the mRNA expression levels of the pro-inflammatory cytokines IL-6 and TNF-α in spleen tissues of mice were analyzed by RT-qPCR. As shown in Figure 1A, IL-6 expression was analyzed across the treatment groups. The inclusion of PEI25k in liposomal formulations enhanced DNA delivery and immune activation (Lu et al. 2020). PEI25k, a cationic polymer, promotes endosomal escape and transfection efficiency (Shen et al. 2018; Dai et al. 2020); it also activates innate immune sensors such as TLRs, leading to the increased production of cytokines, including IL-6 (Jia et al. 2013). The lipid DOTAP further contributed by forming stable lipoplexes with DNA and facilitating antigen uptake by dendritic cells (Miatmoko et al. 2023). Cholesterol stabilized the lipid bilayer and modulated immune responses by reducing membrane fluidity and cytotoxicity associated with PEI25k, thereby attenuating TLR-mediated IL-6 production (Song et al. 2012; Pasarin et al. 2023). According to previous studies, IL-6 – released by macrophages, dendritic cells, and T cells – functions as an inflammatory mediator following antigen exposure and serves as a crucial link between the innate and adaptive immune system by driving the maturation of naïve CD4^+^ T cells into effector lineages such as Th17 cells (Scheller et al. 2011; Tanaka et al. 2014). Notably, the lowest IL-6 expression was observed in the PEI25k : DOTAP : cholesterol (2 : 1 : 1) + DNA group, with a fold change of 0.17 ± 0.01 (*p* < 0.05), suggesting effective modulation of inflammatory responses and reduced risk of excessive immune activation.

Next, we examined TNF-α expression levels across all treatment groups (Figure 1B). Among the tested adjuvants, PEI25k showed a dual role in TNF-α modulation. As a polycationic polymer, PEI25k promotes DNA condensation and endosomal escape, thereby enhancing antigen presentation. The lipid DOTAP also contributed to the regulation of TNF-α expression. DOTAP can stimulate antigen uptake and enhance endosomal escape. Moreover, as a cationic lipid, it can also stimulate innate immune sensors such as pattern recognition receptors (PRRs), for example, TLR2 and TLR4, resulting in increased TNF-α production (Tretiakova and Vodovozova 2022). Cholesterol, a neutral helper lipid, is essential for regulating the membrane fluidity and stability of liposomal carriers (Briuglia et al. 2015; Tada et al. 2015; Kaddah et al. 2018; Wang et al. 2024). In addition to its structural benefits, cholesterol can influence immune signaling, which affects the clustering and activation of TNF receptor-associated pathways (Garcia et al. 2023). TNF-α showed the lowest expression in the PEI25k : DOTAP : cholesterol (2 : 1 : 1) + DNA group, with a fold change of 0.25 ± 0.01 (*p* < 0.05), indicating minimal systemic inflammation. This aligns with the findings of previous studies, which showed that TNF-α, an early-response cytokine produced by activated macrophages, mediates inflammation, leukocyte recruitment, and apoptosis during infection or immunization (Silva et al. 2019; Schultheiß et al. 2022; Chen et al. 2022).

In the present study, the suppression of both IL-6 and TNF-α indicates that the optimized liposomal formulation not only facilitates antigen delivery but also contributes to controlled immune activation. Taken together, these findings demonstrate that the PEI25k : DOTAP : cholesterol (2 : 1 : 1) formulation exhibits strong immunomodulatory potential by downregulating key inflammatory cytokines involved in early immune responses. This observation supports its utility as an effective adjuvant system for DNA vaccines targeting SARS-CoV-2 and potentially other infectious pathogens.

### Comparative phenotypic profiling of immune cells

To assess the immune cell populations induced by liposome-based adjuvant on SARS-CoV-2 spike protein-encoding DNA, flow cytometry analysis was performed on peritoneal exudate cells collected from mice. Leukocyte subsets were identified using fluorescent-labeled antibodies targeting the following cell surface markers: CD4^+^, CD8^+^, CD11b^+^, CD14^+^, and CD45^+^. These markers, commonly referred to as clusters of differentiation (CD), serve as essential tools in immunophenotyping, as they mediate signal recognition, antigen presentation, and intercellular communication across the innate and adaptive branches of the immune system (Kalina et al. 2019; Delmonte and Fleisher 2019). Previous studies have revealed that CD4^+^ and CD8^+^ T lymphocytes are the primary components of the adaptive immune system, CD4^+^ helper T cells support the activity of antigen-presenting cells (APCs) and stimulate B cell responses, and CD8^+^ cytotoxic T lymphocytes (CTLs) target and destroy virus-infected cells by releasing granzyme and perforin (WØrzner et al. 2021).

In COVID-19, several immune cell surface markers are closely associated with IL-6- and TNF-α-mediated inflammation. CD4^+^ T cells contribute to the elevated levels of IL-6 and TNF-α through Th1/Th17 pathway activation (Kalina et al. 2019; Merad and Martin 2020; Schultheiß et al. 2022), while CD8^+^ T cells contribute to TNF-α secretion and can exacerbate tissue damage (Iwasaki and Medzhitov 2015; Kalina et al. 2019). CD11b^+^ myeloid cells, including macrophages and neutrophils, are the significant sources of these cytokines during cytokine storms (Schmid et al. 2018). CD14^+^ monocytes are also upregulated in severe cases and promote pro-inflammatory cytokine release following viral sensing (Wu et al. 2019; Ciesielska et al. 2021). Additionally, CD45^+^ leukocyte infiltration correlates with the elevated levels of IL-6 and TNF-α in inflamed tissues, indicating widespread immune activation (Courtney et al. 2019; Kalina et al. 2019).

**Figure 2:**
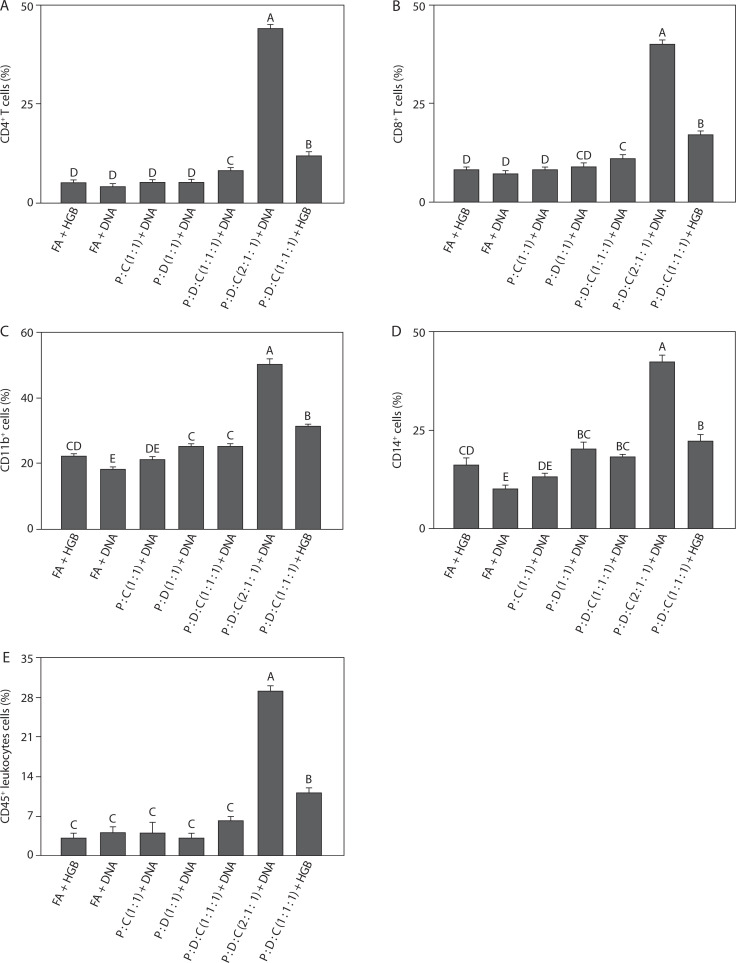
Phenotypic characterization of immune cells induced by the SARS-CoV-2 spike protein-encoding DNA vaccine with the liposome-based adjuvant. Mouse exudate peritoneal cells were labeled with monoclonal antibodies for detecting (**A**) CD4+ T cells, (**B**) CD8+ T cells, (**C**) CD11b+ cells, (**D**) CD14+ cells, and (**E**) CD45+ leukocytes. The labeled cells were analyzed by Attune flow cytometry, and the viability of the cell population was determined by 7-AAD staining. Different letters indicate significant differences between the groups (p < 0.05; n = 3)

The PEI25k : DOTAP : cholesterol (2 : 1 : 1) + DNA group induced the highest percentages of T lymphocytes among all the groups. As shown in Figures 2A and 2B, the percentages of CD4^+^ T (44.00 ± 0.50, *p* < 0.05) and CD8^+^ T cells (40.00 ± 1.00, *p* < 0.05) indicated strong activation of the adaptive immune system. This group also exhibited significantly elevated expression of CD11b^+^ (50.00 ± 2.00) and CD14^+^ cells (42.00 ± 2.00) (*p* < 0.05), which are commonly associated with cells of the myeloid lineage, including monocytes, macrophages, and antigen-presenting dendritic cells (Figures 2C and 2D). CD11b^+^, an integrin molecule, is vital for mediating leukocyte attachment and movement during inflammatory responses. CD14^+^ functions as a co-receptor for LPS recognition, facilitating innate immune activation through TLR4 signaling (Newton and Dixit 2012; Silva et al. 2019). High levels of these markers suggest that PEI25k : DOTAP : cholesterol (2 : 1 : 1) + DNA not only stimulated T cell responses but also activated APCs crucial for initiating the immune response. This activation is particularly important during SARS-CoV-2 infection, as effective antigen presentation is necessary to elicit a strong immune defense targeting the viral spike protein (Merad and Martin 2020; Karwaciak et al. 2021).

As reported previously, PEI25k improves antigen delivery and facilitates endosomal escape; this stimulates CD4^+^ and CD8^+^ T cells and activates CD11b^+^, CD14^+^, and CD45^+^ immune cells through TLR signaling pathways, thereby elevating IL-6 and TNF-α levels, which may lead to excessive inflammation in COVID-19 (Song et al. 2012; Lu et al. 2020; Dai et al. 2020; Dhall et al. 2021). Similarly, DOTAP enhances the internalization of antigens and their presentation through both MHC class I and II pathways, promoting CD4^+^ and CD8^+^ T cell responses while activating CD11b^+^, CD14^+^, and CD45^+^ cells; this also results in elevated pro-inflammatory cytokine levels (Tada et al. 2015; Tretiakova and Vodovozova 2022). Cholesterol plays a regulatory role by stabilizing lipid bilayers and modulating lipid rafts involved in TCR signaling, helping to balance CD4^+^ and CD8^+^ activation and reduce excessive IL-6 and TNF-α secretion from CD11b^+^, CD14^+^, and CD45^+^ cells by preventing overactivation of innate immune pathways (Pasarin et al. 2023; Garcia et al. 2023; Wang et al. 2024). CD45, a leukocyte marker, is essential for T and B cell receptor signaling, and its upregulation reflects general immune activation; thus, its modulation by liposomal components influences the overall inflammatory profile (Courtney et al. 2019). During SARS-CoV-2 infection, the regulation of these immune markers is essential to balance hyperinflammation prevention with the development of effective antiviral immunity following vaccination (Gómez-Rial et al. 2020).

Moreover, the percentages of CD45^+^ leukocytes (29.00 ± 1.00, *p* < 0.05) support the observation of recruitment and activation of a broad range of leukocytes (Figure 2E). CD45 is found on the majority of hematopoietic-derived cells; this marker is essential for regulating T and B cell receptor signaling and serves as an indicator of general leukocyte activation (Amon et al. 2023). Taken together, the increased expression of both innate (CD11b^+^ and CD14^+^ cells) and adaptive (CD4^+^ and CD8^+^ T cells) markers in the PEI25k : DOTAP : cholesterol (2 : 1 : 1) + DNA group indicates an integrated immune activation, supporting the adjuvant potential of this liposomal formulation in DNA vaccine delivery. Recent findings indicate that CD45^+^ leukocytes are the key contributors to the immune response observed in COVID-19-related inflammation, with their activation strongly linked to elevated IL-6 and TNF-α levels in patients with severe disease (Korn and Hiltensperger 2021; Schultheiß et al. 2022; Jin 2020). PEI25k enhances immune cell activation by promoting DNA delivery and TLR signaling, indirectly increasing CD45^+^ cell activation and contributing to pro-inflammatory cytokine production (Lu et al. 2020). Similarly, DOTAP enhances antigen uptake and stimulates CD45^+^ monocytes and dendritic cells, further amplifying IL-6 and TNF-α expression (Tada et al. 2015). Cholesterol modulates lipid raft organization and stabilizes immune cell membranes, helping regulate CD45-mediated signaling and reducing the risk of excessive cytokine release from CD45^+^ cells (Garcia et al. 2023). Taken together, these components distinctly influence CD45^+^ cell behavior and downstream inflammatory responses, highlighting the importance of balanced liposomal formulation in vaccine design.

### Density of T cell-associated double-positive immune subsets

**Figure 3:**
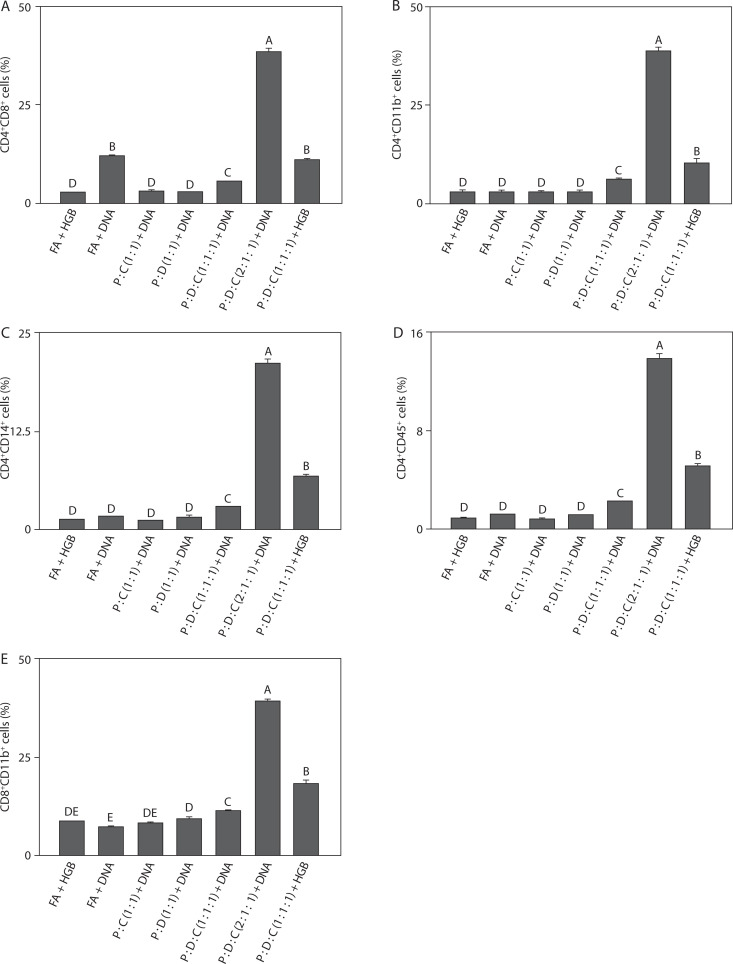
Density of double-positive immune cells induced by the SARS-CoV-2 spike protein-encoding DNA vaccine with the liposome-based adjuvant. Mouse exudate peritoneal cells were labeled with monoclonal antibodies for (**A**) CD4+CD8+ cells, (**B**) CD4+CD11b+ cells, (**C**) CD4+CD14+ cells, (**D**) CD4+CD45+ cells, and (**E**) CD8+CD11b+ cells. The labeled cells were analyzed by Attune flow cytometry, and the viability of the cell population was determined by7-AAD staining. Different letters indicate significant differences between the groups (p < 0.05; n = 3)

To compare baseline immune activation, flow cytometry analysis was performed on the liposomal formulations. The PEI25k : DOTAP : cholesterol (2 : 1 : 1) + DNA group showed the highest cell population among all groups. As shown in Figure 3A, the CD4^+^CD8^+^ cell population (38.3 ± 1.16, *p* < 0.05) indicated activation of the adaptive immune system. This is consistent with previous findings, wherein PEI25k enhances antigen delivery, leading to the induction of CD4^+^ and CD8^+^ T cell responses, and cholesterol contributes to the balanced activation of both cell types; this observation aligns with earlier studies indicating that an effective SARS-CoV-2 vaccine largely depends on strong CD4^+^ and CD8^+^ T cell-mediated immunity (Merad and Martin 2020). Figure 3B depicts the cell population of CD4^+^CD11^+^ (38.64 ± 1.18, *p* < 0.05); as shown in this figure, the stimulation of CD11b^+^ immune cells through TLR signaling by PEI25k elevates inflammation due to elevated pro-inflammatory IL-6 and TNF-α levels, consistent with the findings that SARS-CoV-2 antigens can activate CD11b^+^ myeloid cells to drive inflammatory cytokine production (Shen et al. 2018; Li et al. 2018; Schmid et al. 2018). In addition to the cell population of CD4^+^CD14^+^ (21.05 ± 0.53, *p* < 0.05), which shows a significant increase in lymphocyte and macrophage populations across all groups (Figure 3C). According to previous studies, the innate and adaptive immune activation is likely driven by efficient antigen delivery and presentation through the PEI-based liposomal system; this aligns with earlier reports that SARS-CoV-2 infection and vaccination induce CD14^+^ monocytes that coordinate inflammatory and antiviral responses (Yan and Huang 2007; Ordóñez-Gutiérrez et al. 2015; Sengupta et al. 2022). As shown in Figure 3D, the cell population of CD4^+^CD45^+^ (13.77 ± 0.42, *p* < 0.05) indicated activation of a broad range of leukocytes. This observation suggests that the formulation effectively promotes expansion of immune cell repertoire and facilitates sustained adaptive signaling; this is consistent with the role of PEI25k in enhancing DNA delivery and antigen presentation and highlights the critical contribution of CD45^+^ leukocytes to SARS-CoV-2 immunity and vaccine responsiveness (Song et al. 2012; Negari et al. 2025). Figure 3E also showed a marked elevation in the CD8^+^CD11b^+^ cell population (39.17 ± 0.50, *p* < 0.05). This elevation reflects a strong cytotoxic response coupled with myeloid activation, supporting higher IL-6 and TNF-α levels; this finding agrees with previous evidence that cytotoxic T-cell and CD11b^+^ myeloid activation are the key features of effective SARS-CoV-2 vaccine-induced immunity (Tanaka et al. 2014; Karwaciak et al. 2021). These results highlight the potency of PEI25k : DOTAP : cholesterol in promoting cytotoxic T-cell response and stimulating the innate immune system.

Taken together, the findings from Figures 3A–E show that PEI25k : DOTAP : cholesterol (2 : 1 : 1) + DNA consistently induced the highest immune cell activation across multiple T cell subsets, including CD4^+^CD8^+^, CD4^+^CD11b^+^, CD4^+^CD14^+^, CD4^+^CD45^+^, and CD8^+^CD11b^+^ T cell populations. This broad activation profile reflects both adaptive and innate immune stimulation, characterized by enhanced T-cell proliferation, leukocyte engagement, macrophage recruitment, and cytotoxic activity (Iwasaki and Medzhitov 2015; Pinti et al. 2016; Zimmermann and Curtis 2019). The results strongly support the application of PEI25k : DOTAP : cholesterol as an effective liposomal adjuvant formulation, capable of promoting robust antigen delivery and eliciting balanced immune responses critical for enhancing the efficacy of spike-protein encoding SARS-CoV-2 DNA vaccine. The consistent activation of multiple immune subsets also highlights the formulation’s ability to enhance overall immunogenicity, which is essential for generating long-lasting and effective immune protection against SARS-CoV-2.

### Density of myeloid-associated double-positive immune subsets

**Figure 4:**
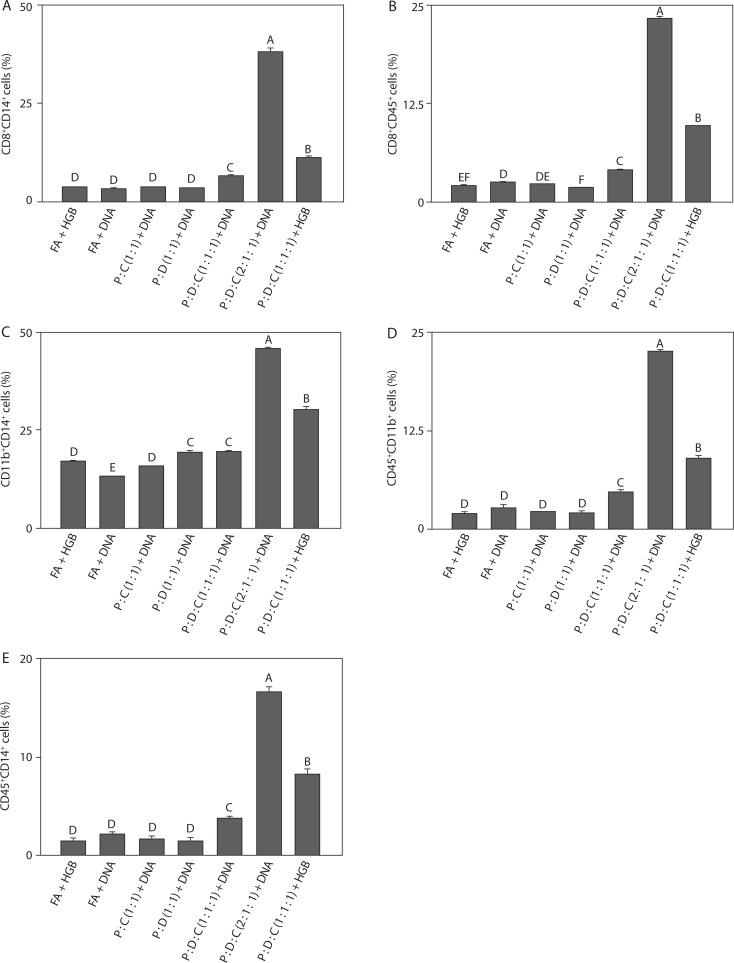
Density of double-positive immune cells induced by the SARS-CoV-2 spike protein-encoding DNA vaccine with the liposome-based adjuvant. Mouse exudate peritoneal cells were labeled with monoclonal antibodies for (**A**) CD8+CD14+ cells, (**B**) CD8+CD45+ cells, (**C**) CD11b+CD14+ cells, (**D**) CD45+CD11b+ cells, and (**E**) CD45+CD14+ cells. The labeled cells were analyzed by Attune flow cytometry, and the viability of the cell population was detected by 7-AAD staining. Different letters indicate significant differences between the groups (p < 0.05; n = 3)

To assess immune responses across all groups, the liposomal formulation was subjected to flow groups. As shown in Figure 4A, the cell population of CD8^+^CD14^+^ (38.00 ± 1.00, *p* < 0.05) was the highest. This increase reflects strong cytotoxic T-cell activity accompanied by monocyte/macrophage recruitment, indicating effective coordination between adaptive and innate immune responses; this finding is consistent with reports that SARS-CoV-2 vaccines rely on CD8^+^ T cells and monocyte subsets for antiviral defense (Zimmermann and Curtis 2019; Garcia et al. 2023). As shown in Figure 4B, the cell population of CD8^+^CD45^+^ (23.26 ± 0.29, *p* < 0.05) indicates CD45 expression on cytotoxic T cells, thus suggesting robust leukocyte activation and supporting the role of the PEI25k-based formulation in promoting broad immune signaling and sustained antigen-specific responses; this observation agrees with previous findings that CD45^+^ cytotoxic T cell activity is a hallmark of effective SARS-CoV-2 vaccine-induced immunity (Song et al. 2012; Courtney et al. 2019). As depicted in Figure 4C, the cell population of CD11b^+^CD14^+^ (45.56 ± 0.74, *p* < 0.05) reflects strong co-expression, which highlights enhanced activation of myeloid-derived cells, particularly monocytes and macrophages – key drivers of inflammatory processes and release of cytokines such as IL-6 and TNF-α; this result aligns with previous evidence that SARS-CoV-2 infection and vaccination trigger CD11b^+^CD14^+^ myeloid activation as part of immune regulation (Schmid et al. 2018; Wu et al. 2019; Ciesielska et al. 2021). Figure 4D shows a significant increase in CD45^+^CD11b^+^ cell population (22.50 ± 0.35, *p* < 0.05). This result suggests that helper T-cell activation was closely linked with myeloid cell engagement, indicating that the liposomal formulation effectively bridges adaptive and innate immune pathways, which is consistent with previous studies showing that SARS-CoV-2 vaccines enhance interactions between CD45^+^ lymphocytes and CD11b^+^ myeloid cells to sustain immunity (Vašíček and Balazi 2015; Courtney et al. 2019). Finally, as illustrated in Figure 4E, the cell population of CD45^+^CD14^+^ (16.63 ± 0.47, *p* < 0.05) underscores broad leukocyte involvement with strong monocyte/macrophage participation, further confirming the ability of this adjuvant system to amplify inflammatory and antigen-presenting cell activity; this observation agrees with previous reports that CD14^+^ monocytes are the key contributors to immune activation triggered by SARS-CoV-2 vaccination (Kalina et al. 2019; Rosales 2020).

Taken together, the results described in Figures 4A–E demonstrate that PEI25k : DOTAP : cholesterol (2 : 1 : 1) + DNA consistently enhanced immune activation across multiple immune cell subsets, such as CD8^+^ cytotoxic T lymphocytes, CD4^+^ helper T cells, and myeloid-derived populations. The significant increase in CD8^+^CD14^+^, CD8^+^CD45^+^, CD11b^+^CD14^+^, CD4^+^CD11b^+^, and CD45^+^CD14^+^ populations indicates an integrated stimulation of both adaptive and innate immune responses. These findings confirm that the PEI25k-based liposomal adjuvant not only promotes antigen-specific T cell responses but also strengthens the activity of monocytes and macrophages, resulting in the elevated production of pro-inflammatory cytokines (Lu et al. 2020; Rosales 2020). Altogether, this broad immune engagement highlights the potency of PEI25k : DOTAP : cholesterol as a versatile adjuvant for SARS-CoV-2 spike protein-encoding DNA vaccine formulations. Moreover, the ability to simultaneously activate diverse immune cell subsets emphasizes the strong immunogenicity of this adjuvant, which is essential for generating durable and protective antiviral immunity.

## Discussion

The effectiveness of vaccines can be determined by quantifying the expression levels of pro-inflammatory cytokines, particularly IL-6 and TNF-α, which contribute substantially to innate immune activation (Tanaka et al. 2014; Arts et al. 2018). However, because innate immunity lacks memory, adaptive immunity is required to achieve long-term protection. Adaptive immunity involves lymphocytes, encompassing T cells activated by APCs and B cells maturing into antibody-secreting plasma cells (Marshall et al. 2018). IL-6 acts as a bridge between innate and adaptive immune responses by driving the maturation of naïve CD4^+^ and CD8^+^ T cells (Scheller et al. 2011), while TNF-α supports the activation of T and B lymphocytes through macrophage- and NK cell-mediated pathways (Silva et al. 2019). In the present study, a DNA vaccine encoding the SARS-CoV-2 spike (S) protein was tested; however, similar to other DNA vaccines, its immunogenicity was found to be limited due to inefficient cellular uptake (Hayat Khan 2013; Smith et al. 2020; Eusébio et al. 2021; Narayanan et al. 2022; Ledesma-Feliciano et al. 2023). To address this concern, we used liposome-based adjuvants to protect the DNA vaccine from degradation, enhance immune responses, and maintain low toxicity (Tretiakova and Vodovozova 2022; Lu and Qi 2021). Liposomes possess strong immunostimulatory potential, good biocompatibility, and high antigen encapsulation capacity (Tretiakova and Vodovozova 2022; Negari et al. 2025; Lu and Qi 2021); moreover, they have been widely utilized as delivery vehicles to stimulate immune responses in SARS-CoV-2 vaccines (Lee et al. 2015). However, because their phospholipid membranes are structurally fragile (Pasarin et al. 2023), stabilizers such as PEI are required to prevent their structural disintegration (Liang et al. 2019; Dai et al. 2020; Sriwidodo et al. 2022; Pasarin et al. 2023). Although PEI25k improves DNA condensation and endosomal escape, it is also cytotoxic. This effect can be mitigated by cholesterol, which stabilizes the membrane and neutralizes excess PEI25k charges (Song et al. 2012; Jia et al. 2013), while DOTAP further enhances DNA complexation and delivery (Miatmoko et al. 2023). Recent vaccine studies also emphasize that adjuvant selection strongly influences cytokine and T cell outcomes; for example, a heat-killed *Caulobacter crescentus*-based adjuvant enhanced mucosal and systemic T cell responses in a SARS-CoV-2 vaccine (Patel et al. 2025). Likewise, novel lipid-based adjuvants could modulate IL-6 and TNF-α levels while sustaining antigen-specific immunity (Harberts et al. 2025); these reports support our findings regarding the importance of well-formulated liposomal composition. Consistent with these mechanisms, our results showed that the PEI25k : DOTAP : cholesterol (2 : 1 : 1) + DNA formulation significantly reduced IL-6 and TNF-α expression levels compared to controls, indicating effective immune modulation (Figure 1). In contrast, the positive controls – Freund’s adjuvant + DNA or HGB – produced persistently high cytokine levels, suggesting uncontrolled inflammation and poor memory response development. The reduction in cytokine expression levels following liposomal formulation administration suggests a more regulated inflammatory response, potentially favoring immune memory formation. This observation aligns with the broader use of PEI as vaccine carriers, as they exhibit good biocompatibility, stability, and low toxicity while facilitating efficient antigen delivery in DNA vaccines (Franck et al. 2021).

Cytokines are key mediators of both inflammation and adaptive immune responses (Hagan and Pulendran 2018). Elevated IL-6 and TNF-α levels reflect immune activation involving both humoral and cellular responses (Bergamaschi et al. 2021). The finding that the PEI25k : DOTAP : cholesterol (2 : 1 : 1) formulation exhibits immunosuppressive effects by reducing TNF-α and IL-6 levels is potentially interesting. This indicates that the formulation could help mitigate the inflammatory adverse effects commonly associated with DNA vaccines. The PEI25k : DOTAP : cholesterol (2 : 1 : 1) formulation also appears to have immunostimulatory effects, as evidenced by an increase in immune cell populations. Our results confirm that liposomal formulations can modulate these cytokines, consistent with reports that spike protein-induced cytotoxicity elevates IL-6 and TNF-α levels (Mulchandani et al. 2021; Liu et al. 2022; Queiroz et al. 2022; Kao et al. 2022), while liposome-based vaccines mitigate this effect by regulating immune activation (Gregoriadis 2021; Abhyankar et al. 2021; Ober Shepherd et al. 2024). This is particularly relevant because IL-6 overexpression contributes to the cytokine storm syndrome in severe COVID-19 cases (Liang et al. 2020), while TNF-α overexpression is associated with tissue damage and chronic inflammation (Arunachalam et al.). Moreover, SARS-CoV-2 induces macrophages to release IL-6 that drives lymphocyte necrosis; the virus also neutralizes the activity of spleens and lymph nodes through infection of tissue-resident CD169^+^ macrophages, highlighting the importance of vaccine formulations that can control hyperinflammation (Feng et al. 2020). These findings demonstrate that the PEI25k : DOTAP : cholesterol (2 : 1 : 1) + DNA formulation robustly activates innate immunity, modulates inflammation, and promotes adaptive memory formation. These properties demonstrates the potential of PEI25k : DOTAP : cholesterol (2 : 1 : 1) as the most effective liposome-based adjuvant system for a SARS-CoV-2 spike protein-encoding DNA vaccine.

As demonstrated by previous studies, peritoneal exudate cells and splenic cells harbor a rich population of immune markers, such as F4/80^+^ monocytes, which critically contribute to induce the activation of regulatory T cells and initiate systemic immune responses, highlighting their importance in modulating both innate and adaptive immunity following antigen exposure (Cone and Pais 2009). Furthermore, leukocytes, including monocytes, macrophages, dendritic cells, neutrophils, and lymphocytes (NK and T cells), express CD molecules on their surfaces, which are essential for detecting environmental threats and mediating immune cell interactions (Kalina et al. 2019). These CD molecules are commonly used as cell surface markers to identify and quantify leukocyte populations and specific lymphocyte subsets by using fluorochrome-labeled antibodies and flow cytometry. Thus, detecting the presence of these leukocytes is crucial in studies aiming to identify immune responses following vaccination. Accordingly, the present study analyzed leukocyte and lymphocyte marker expression from mouse peritoneal exudates following the administration of various DNA-liposome vaccine formulations. Flow cytometry was used to detect five surface markers, namely CD4, CD8, CD11b, CD14, and CD45, which were chosen to assess both innate and adaptive immune responses. These leukocytes express surface molecules (Kalina et al. 2019), commonly used as cell surface markers to identify and quantify leukocyte populations and specific lymphocyte subsets associated with cellular function using fluorochrome-labeled antibodies and flow cytometry.

The results of flow cytometry analysis (Figure 2) showed significant leukocyte activation in the PEI25k : DOTAP : cholesterol (2 : 1 : 1) + DNA group, indicating that antigen exposure stimulated immune cell production (Kalina et al. 2019; Kostinov et al. 2020). CD4 serves as a marker for lymphocytes, particularly T helper cells, which are essential for adaptive immunity (WØrzner et al. 2021). High CD4 expression in this group suggests the induction of lymphocytes, including T cells, thus reflecting an active immune response; this finding is consistent with the immunostimulatory effects of liposomal adjuvants in SARS-CoV-2 vaccines (Wang et al. 2019; Abhyankar et al. 2021; Ober Shepherd et al. 2024). CD11b^+^ and CD14^+^ cells reflect the activation of innate immune cells, particularly myeloid cells such as macrophages, neutrophils, and dendritic cells. In the present study, the population of CD11b^+^-expressing cells was the highest in the PEI25k : DOTAP : cholesterol (2 : 1 : 1) + DNA group, indicating the activation of macrophages and APCs, which are crucial for initiating immune responses (Lakschevitz et al. 2016; Buoninfante et al. 2024). This observation agrees with prior studies showing increased CD11b expression following SARS-CoV-2 vaccination (Vitallé et al. 2022; Acosta-Altamirano et al. 2022). The expression of CD14, a TLR4 co-receptor, also increased, suggesting the activation of phagocytes in response to vaccine antigen recognition (Newton and Dixit 2012). Macrophages and dendritic cells, activated shortly following antigen exposure, act as primary effectors of innate immunity (Newton and Dixit 2012); this suggests that the PEI25k : DOTAP : cholesterol (2 : 1 : 1) formulation successfully triggered innate immunity in mice. The population of CD45^+^-expressing leukocytes was also high in the PEI25k : DOTAP : cholesterol (2 : 1 : 1) + DNA group (Figure 3). Because CD45 is a marker for lymphocytes (Kalina et al. 2019; Amon et al. 2023), these findings support previous reports that SARS-CoV-2 DNA vaccines can induce lymphocyte formation essential for adaptive immunity (Silva et al. 2019).

In addition to cell characterization, dual-marker analysis was conducted to assess leukocyte populations in each treatment group. The findings showed that PEI25k : DOTAP : cholesterol (2 : 1 : 1) + DNA elicited the strongest immune activation across multiple double-positive subsets, including CD4^+^CD8^+^, CD4^+^CD11b^+^, CD4^+^CD14^+^, CD4^+^CD45^+^, and CD8^+^CD11b^+^ cells (Figure 3); this observation is consistent with previous reports that SARS-CoV-2 vaccines require coordinated activation of both T-cell and myeloid compartments for optimal protection (Shim et al. 2010; Nugent 2022). The increase in CD4^+^CD8^+^ cell populations reflects balanced stimulation of helper and cytotoxic T cell pathways, highlighting the crucial role of lymphocytes in memory formation (Rabaan et al. 2021). For instance, Weiskopf et al. (2020) found that SARS-CoV-2-specific CD4^+^ and CD8^+^ T cells emerge early and their populations expand during infection, while Grifoni et al. (2020) showed that, in most convalescent patients, broad T cell responses targeting spike, membrane, and nucleocapsid proteins are generated. Braun et al. (2020) also identified spike-reactive CD4^+^ T cells in both infected patients and unexposed donors, and Sałkowska et al. (2020) observed that SARS-CoV-2 antigens induce strong Th1-type cytokine secretion in human T cells, confirming that SARS-CoV-2 consistently elicits robust helper and cytotoxic T cell responses; these results highlight the essential contribution of T cells to viral clearance and long-term protective immunity. According to a previous study, CD8^+^ T cells isolated from mouse spleen were found to perform essential functions in regulating immune responses, particularly in suppressing delayed-type hypersensitivity through mechanisms associated with immune tolerance (D’Orazio and Niederkorn 1998). This finding supports the relevance of splenic CD8^+^ populations as key effectors in modulating antigen-specific responses following vaccination, consistent with evidence that SARS-CoV-2-specific CD8^+^ T cells contribute to virus elimination and long-term immunity (Iwasaki and Medzhitov 2015; Silveira et al. 2021; Tiyo et al. 2021). The increase in CD4^+^CD11b^+^ and CD4^+^CD14^+^ population subsets suggests enhanced T cell interaction with myeloid-derived cells, facilitating effective antigen presentation and inflammatory signaling. Likewise, the considerable expansion of CD4^+^CD45^+^ and CD8^+^CD11b^+^ subsets indicates broad leukocyte involvement and enhanced cytotoxic activity, supporting effective bridging of innate and adaptive immune responses; this observation further emphasizes the importance of balanced leukocyte activation in achieving durable immunogenicity against SARS-CoV-2 (Merad and Martin 2020; Choudhary et al. 2024).

Complementary results presented in Figure 4 further confirmed the potency of this formulation, with a marked increase in CD8^+^CD14^+^, CD8^+^CD45^+^, CD11b^+^CD14^+^, CD4^+^CD11b^+^, and CD45^+^CD14^+^ cells. These profiles reflect robust cytotoxic T cell activation and strong monocyte and macrophage response, which are critical for antigen uptake, processing, and cytokine release; moreover, the observed patterns are consistent with the findings that effective SARS-CoV-2 vaccines require coordinated activation of cytotoxic and myeloid compartments (Gómez-Rial et al. 2020; McElvaney et al. 2020). The integration of T cell and myeloid activation suggests that PEI25k : DOTAP : cholesterol effectively promotes immune synergy, driving both cellular cytotoxicity and innate inflammatory responses, including IL-6 and TNF-α production; this is in line with previous results highlighting that SARS-CoV-2-induced protection is strongly associated with T cell and cytokine-mediated immunity (Ye et al. 2020; Darif et al. 2021; Kutumbetov et al. 2024). Mechanistically, the enhanced immunogenicity observed in the PEI25k : DOTAP : cholesterol (2 : 1 : 1) + DNA group can be attributed to the complementary functions of its components. PEI25k is a cationic polymer that efficiently condenses DNA, facilitating cellular uptake and endosomal escape, while cholesterol contributes to liposome stability and membrane fusion, ensuring efficient antigen delivery. Collectively, this balanced formulation enhances antigen presentation and immune cell activation while minimizing cytotoxicity that may occur due to a high PEI concentration, further emphasizing its potential as a vaccine adjuvant for SARS-CoV-2 DNA vaccine development (Haensler 2010; Liang et al. 2020; Ndwandwe and Wiysonge 2021; Zhao et al. 2023). In summary, liposome-based adjuvants exhibit strong immunogenicity, which can enhance the efficacy of SARS-CoV-2 spike protein-encoding DNA vaccines, thereby ensuring more effective induction of protective and long-lasting immune responses.

## Conclusions

The present study demonstrated that liposome-based adjuvant formulations incorporating PEI25k, DOTAP, and cholesterol effectively enhanced the immune response induced by a SARS-CoV-2 spike protein-encoding DNA vaccine. The formulation promoted both innate and adaptive immunity, as evidenced by elevated expression of pro-inflammatory cytokines (IL-6 and TNF-α) and robust activation of leukocytes. Specifically, the populations of CD4^+^ T cells, CD8^+^ T cells, CD11b^+^ myeloid cells, CD14^+^ monocytes/macrophages, and CD45^+^ leukocytes were significantly increased, indicating the engagement of a broad range of immune cells across critical cellular subsets. These results highlight the ability of liposomal carriers to improve DNA delivery, enhance antigen presentation, and balance pro-inflammatory signaling while minimizing cytotoxicity. Overall, the findings indicate that liposome-based adjuvants are promising platforms, in terms of safety and efficacy, for developing DNA vaccines against SARS-CoV-2. Notably, the PEI25k : DOTAP : cholesterol (2 : 1 : 1) formulation shows strong immunogenicity, which improves the efficacy of the SARS-CoV-2 spike protein-encoding DNA vaccine, thus confirming that this formulation could be used as a promising candidate adjuvant in future vaccine development.
